# Safety and effectiveness of a novel dielectric mapping system: one-year, two chinese centers experiences

**DOI:** 10.1186/s12872-022-02790-8

**Published:** 2022-08-03

**Authors:** Lei Ding, Xiao Huang, Cong Dai, Hongda Zhang, Sixian Weng, Fengyuan Yu, Yingjie Qi, Shu Zhang, Ruizheng Shi, Min Tang

**Affiliations:** 1 State Key Laboratory of Cardiovascular Disease, Department of Cardiology, Cardiovascular Institute, Fuwai Hospital, National Center for Cardiovascular Diseases, Chinese Academy of Medical Sciences, and Peking Union Medical College, No.167 North Lishi Rd, Xicheng, Beijing, 100037 People’s Republic of China; 2 Department of Cardiovascular Medicine, Xiangya Hospital, Central South University, Changsha, Hunan People’s Republic of China; 3 Department of Health Policy and Management, School of Public Health, Peking University Health Science Center, Beijing, 100191 People’s Republic of China

**Keywords:** Dielectric mapping system, Supraventricular tachycardia, Atrial fibrillation, Catheter ablation, Safety, Outcomes

## Abstract

**Background:**

The KODEX-EPD system is a novel, dielectric three-dimensional mapping system. We aim to illustrate the feasibility, safety, and outcomes of ablation using the KODEX-EPD system.

**Methods:**

A total of 272 patients with supraventricular arrhythmias were enrolled and underwent catheter ablation using the KODEX-EPD system from October 2020 to July 2021. The feasibility, safety, and ablation outcomes were analyzed.

**Results:**

Of the enrolled patients, 15 (5.4%) had atrial tachycardia (AT), 88 (31.4%) had atrioventricular reentrant tachycardia (AVRT), 141 (50.4%) had atrioventricular nodal reentrant tachycardia (AVNRT), 34 (12.1%) had atrial fibrillation (AF), and 9 (3.2%) had atrial flutter (AFL). All AF patients included were first-do-pulmonary vein isolation (PVI); there were 26 paroxysmal AF and 8 persistent AF. All patients achieved immediate success of ablation. The mean follow-up duration was 11.8 ± 2.4 months. One patient (1.1%) in the AVRT subgroup and two patients (1.4%) in the AVNRT subgroup experienced recurrence. When considering a three-month blanking time, the estimated freedom of AF at one-year post-ablation with and without AADs was 75.7% and 70.4%, respectively. The Kaplan–Meier analysis showed no significant difference in the overall AF recurrence (log-rank; P = 0.931) or AAD-free AF recurrence (log-rank; P = 0.841) between RFCA and cryoablation. One patient had mild pulmonary embolism. None of the patients died or had a cerebrovascular event in the periprocedural period.

**Conclusions:**

This retrospective, two-center study demonstrated that catheter ablation of supraventricular arrhythmias using the KODEX-EPD system is feasible, safe, and effective.

*Trial registration* Retrospectively registered.

**Supplementary Information:**

The online version contains supplementary material available at 10.1186/s12872-022-02790-8.

## Introduction

Radiofrequency catheter ablation (RFCA) is an effective way to treat tachyarrhythmias. The conventional method under fluoroscopy has been gradually replaced by the three-dimensional (3D) mapping system. However, despite advances in ablation devices, RFCA remains a challenging procedure with a long learning curve [1]. Under this circumstance, precise 3D images of the heart and accurate navigation of the catheters are crucial for performing a safe and effective RFCA. Particularly for some complex arrhythmias with advanced ablation strategies, the exact location of specific anatomic structures and their variations are determinants for RFCA.

The KODEX-EPD system is a novel, real-time, in vivo 3D cardiac mapping system that is mainly based on the dielectric characteristics of different tissues. The body surface patches and catheter electrodes could both generate an electric field; when the catheter moves within the electric field, high-resolution 3D images of cardiac anatomy are created without physical contact. This dielectric mapping system could provide not only 3D surface images but also an endocardial surface of the heart which could offer a unique option for operators to design ablation strategies.

In this study, we aim to illustrate the feasibility, safety, and outcomes of ablation using the novel wide-band dielectric mapping system in two Chinese centers.

## Methods

### Study population

This study is a two-center, retrospective study. A total of 272 patients who were diagnosed of arrhythmias during electrophysiological procedures and underwent ablation using the KODEX-EPD system from October 2020 to July 2021 in the Department of Cardiology, Fuwai Hospital and Xiangya Hospital were included in this analysis. Patients were included for documented symptomatic supraventricular tachycardia (SVT, including suspected atrioventricular nodal reentrant tachycardia [AVNRT], atrioventricular reentrant tachycardia [AVRT], isthmus-dependent atrial flutter [AFL] and atrial tachycardia [AT]), “and/or” with documented atrial fibrillation (AF). Exclusion criteria included myocardial infarction within the three months before intervention, left ventricular ejection fraction < 35%, or malignant tumor and severe pulmonary disease. The clinical data, including baseline characteristics, standard 12-lead surface electrocardiograms (ECGs), intracardiac electrograms, three-dimensional (3D) electroanatomic mapping data, imaging data, and follow-up outcomes were documented in the study. All patients signed the informed consent form. Study protocols were approved by the independent ethics committees at each study center and were in accordance with the Declaration of Helsinki.

### KODEX EPD system and 3D mapping

**Fig. 1:**
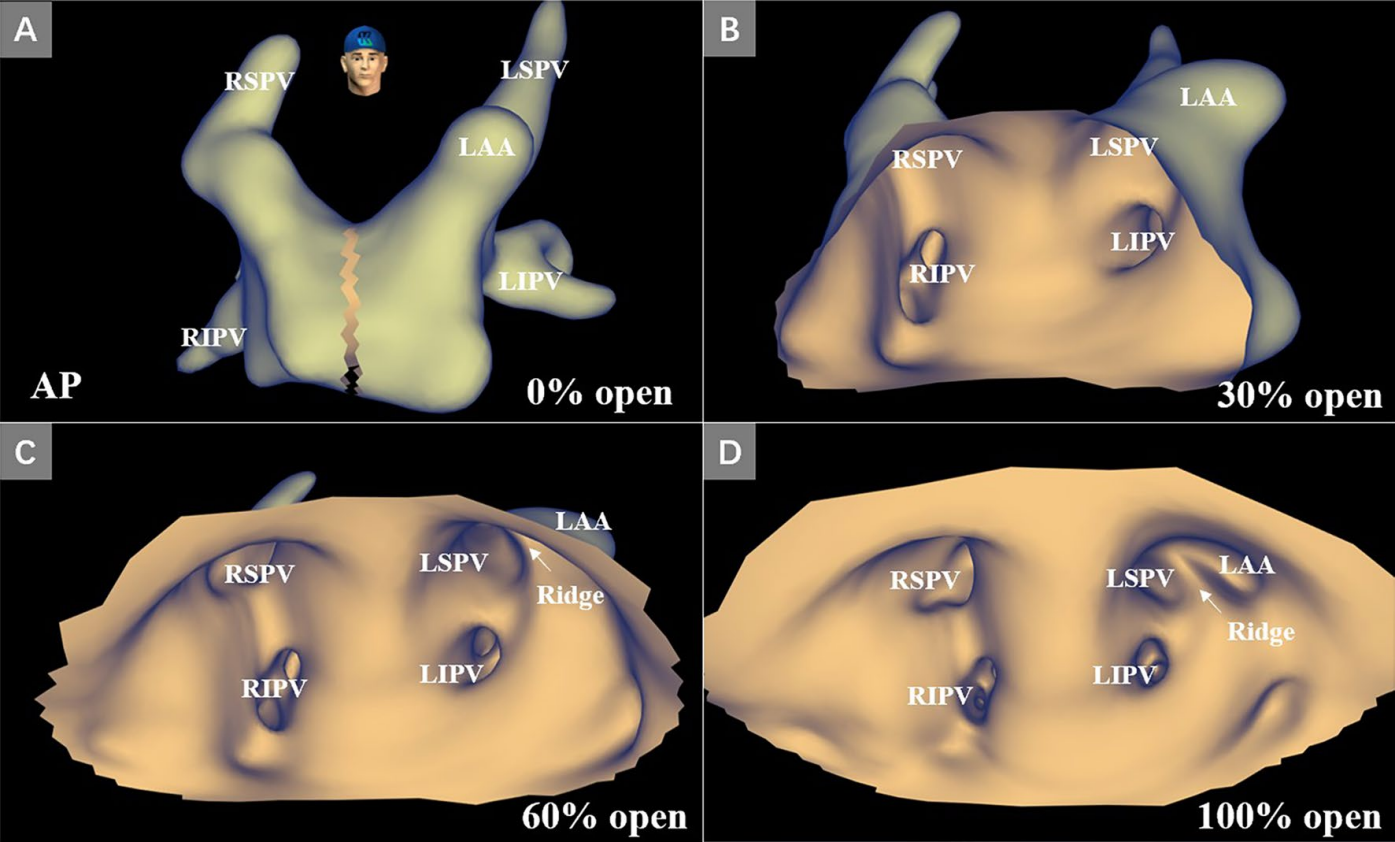
Flow chart of opening the PANO View in a 3D reconstruction of LA. **A** Anteroposterior view of the anatomical 3D reconstruction used to open the PANO View. **B**–**D** Gradual virtual opening of the PANO View. **B** 30% opening; **C** 60% opening; **D** 100% opening. 3D, three-dimensional; AP, anteroposterior view; LIPV, left inferior pulmonary vein; LSPV, left superior pulmonary vein; RIPV, right inferior pulmonary vein; RSPV, right superior pulmonary vein; PANO view, panoramic view

**Fig. 2:**
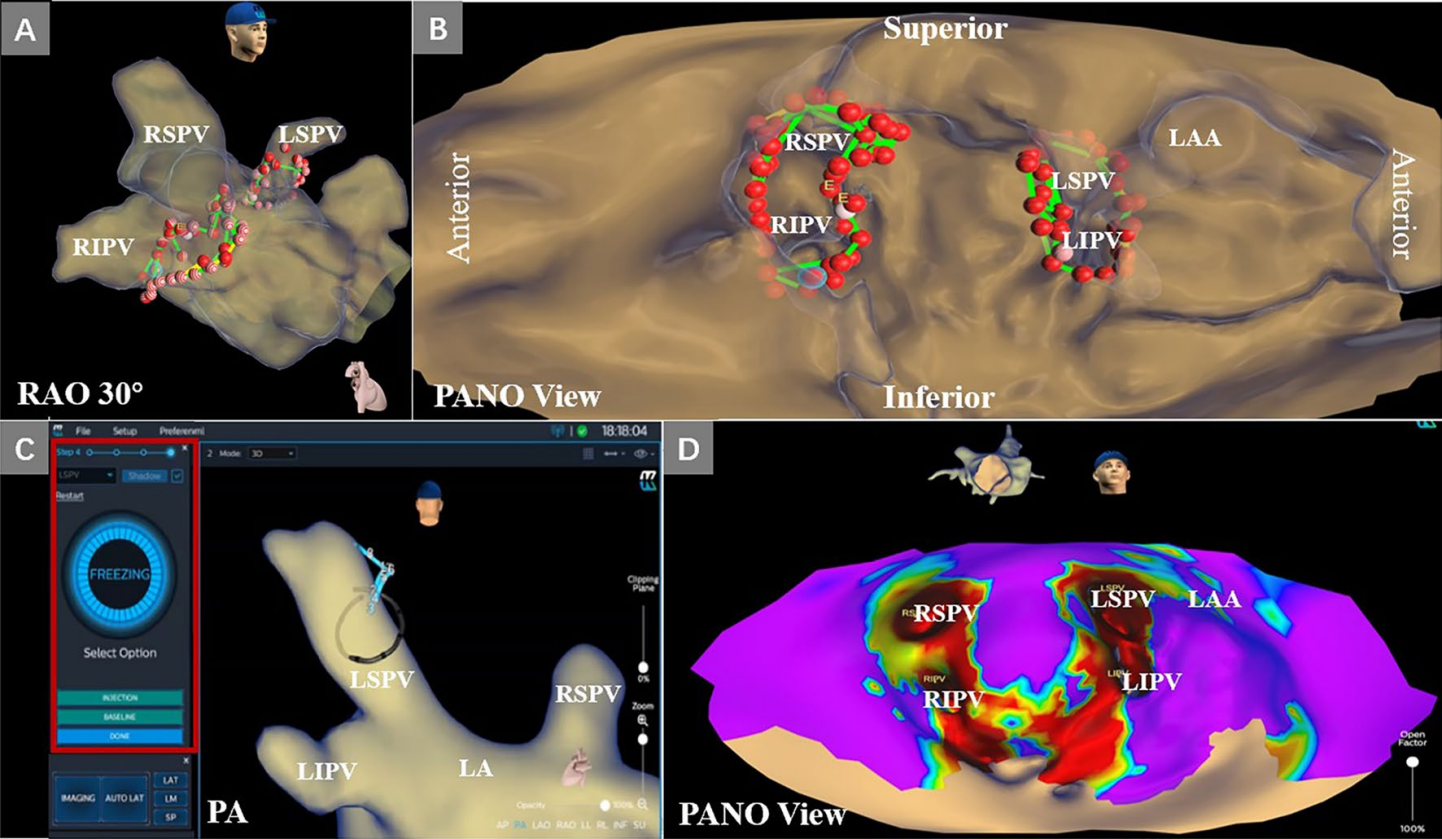
RFCA and cryoablation of AF using the KODEX-EPD system. **A**, **B** A patient with AF underwent RFCA under 3D reconstruction and PANO View. **A** Ablation lines of bilateral PVI; **B** Ablation lines in PANO View. **C**, **D** A patient with AF underwent cryoballoon ablation using occlusion tool software. **C** Freezing phase after checking for gaps using occlusion tool software in LSPV; **D** Voltage mapping of LA after cryoballoon ablation of pulmonary veins in PANO View. Red points represent ablation points; green line segments represent a distance between two ablation points less than 5 mm. 3D, three-dimensional; AF, atrial fibrillation; LA, left atrium; LAA, left atrium appendage; LAO, left anterior oblique; LIPV, left inferior pulmonary vein; LSPV, left superior pulmonary vein; RAO, right anterior oblique; RFCA, radiofrequency catheter ablation; RIPV, right inferior pulmonary vein; RSPV, right superior pulmonary vein; PA, posteroanterior projection; PANO view, panoramic view; PVI, pulmonary vein isolation

**Fig. 3:**
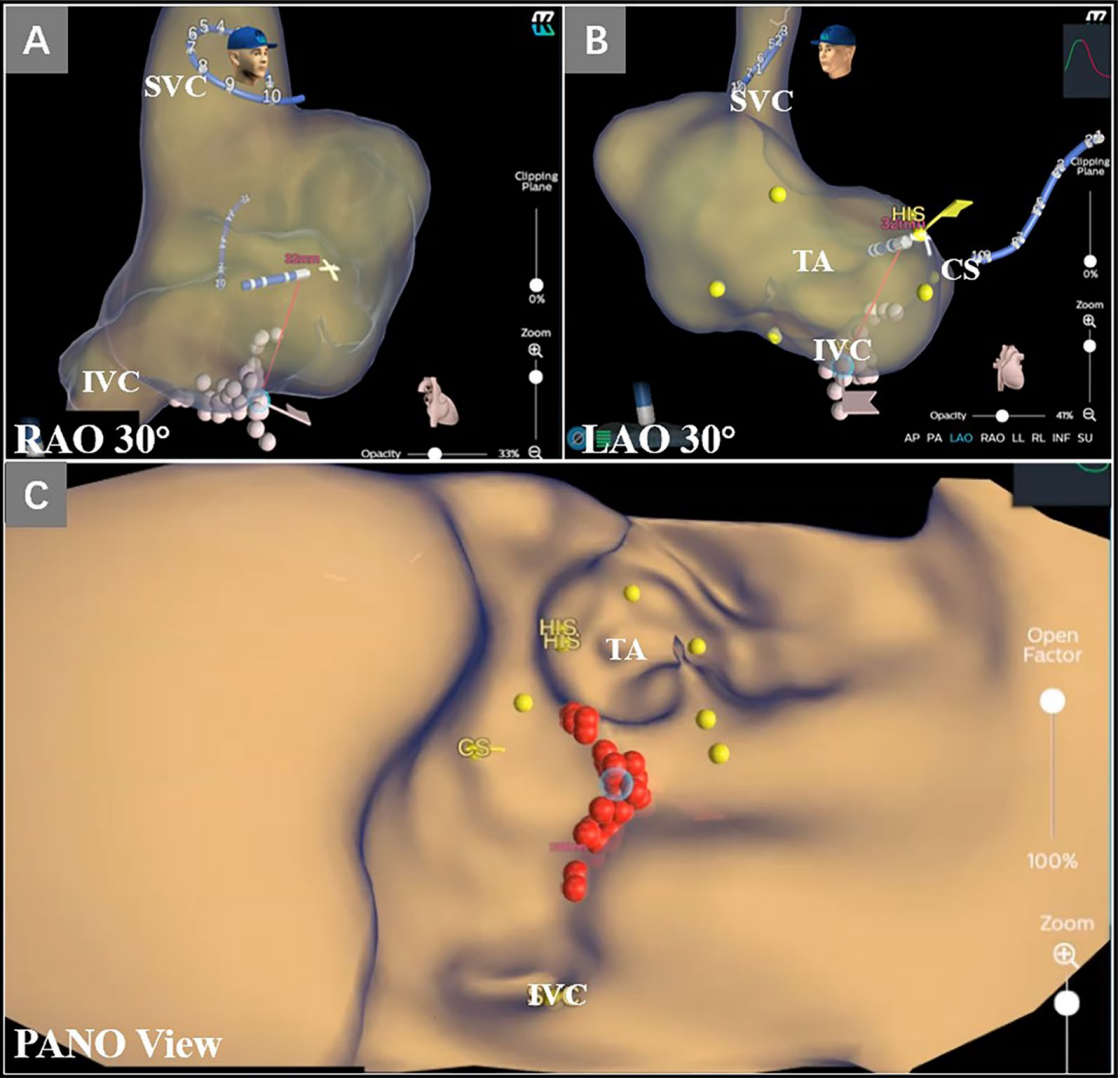
A 52-year-old female patient with AFL underwent RFCA. **A**, **B** Target distribution in RAO and LAO; **C**: Linear ablation applications of tricuspid isthmus in PANO View. AFL, atrial flutter; CS, coronary sinus; HIS, His bundle; IVC, inferior vena cava; LAO, left anterior oblique; RAO, right anterior oblique; RFCA, radiofrequency catheter ablation; PANO view, panoramic view; TA, tricuspid annulus; SVC, superior vena cava

The KODEX EPD (EPD Solutions, Philips, Best, The Netherlands) mapping system uses a novel dielectric energy source. This system receives electrical field transmission and reflection information among patches and catheter electrodes when they are in the cardiac chambers. Different tissues, such as the endocardial surface, coronary sinus, and heart valves, cause different changes in the electrical field. These changes are detected by the system and used to calculate the geometric location of those tissues. With this technique, the KODEX EPD system could accurately detect catheter location and generate high-resolution images without contacting the endocardial surface. It also offers an innovative approach, which is shown as the opened heart virtually across the 3D surface, allowing visualization of the endocardial surface of the cardiac anatomy (Fig. 1). This panoramic view (PANO View) offers a depiction of the endocardial surface and helps to design ablation strategies (Figs. 2B, 3). In addition, the KODEX-EPD system provides a novel tissue-pressure technology (TP, available only in China at the study time) that can monitor the real-time contact force (CF). According to the TP values, the KODEX-EPD system classifies TP into three levels: low TP (< 3 g), normal TP (3–30 g), and high TP (> 30 g). For cryoablation of AF, the KODEX EPD system was equipped with a specific software tool (occlusion tool), which helped to detect the real-time pulmonary vein (PV) occlusion grade (Fig. 2C).

### General electrophysiology study and catheter ablation

All patients discontinued antiarrhythmic drugs (AADs) for at least five half-lives and completed preoperative examinations before the electrophysiological study. Left atrial thrombi were excluded by transesophageal echocardiography one day before ablation. The surface electrocardiogram and bipolar intracardiac electrograms were continuously monitored and stored on a computer-based digital recording system (C. R. Bard, Inc., Lowell, MA). A 6F decapolar steerable catheter (Triguy; APT Medical, CHN) was inserted into the coronary sinus through the left femoral vein. Two standard quadripolar catheters (6F catheter; Triguy; APT Medical, CHN) were placed in the His bundle position and right ventricle through the right femoral vein in patients with supraventricular tachycardia. When needed, left atrial access was attempted by transseptal puncture, and heparin was given. The mapping catheter was advanced via an SL1 or SR0 long sheath if necessary.

Each patient underwent a standard electrophysiology study including programmed stimulation and diagnostic pacing maneuvers [2]. In patients who could not induce tachycardia by a regular procedure, isoproterenol was administered. Radiofrequency energy was delivered through a generator (Stockert, Biosense-Webster, Diamond Bar, CA). The radiofrequency power settings followed the standards of the laboratory, ranging from 20 to 40 W for all patients.

The end points of the procedure were as follows: (1) AVNRT: either a slow pathway blocked or a single atrial echo beat at baseline and during orciprenaline infusion without inducing tachycardia; (2) AVRT: appearance of decremental antegrade and retrograde AV conduction as well as could not induce tachycardia; (3) AFL: bidirectional conduction block confirmed by differential pacing; (4) AF: absence of ostial PV potentials on a circumferential mapping catheter and no atrial capture from multiple pacing sites within the PV ostium. End points had to be fulfilled after a waiting period of 20 minutes after the last ablation. Procedure time was defined as the time from the puncture of the femoral vein to sheath removal.

### RFCA procedure of SVT

Diagnosis of SVT were based on standard electrophysiological criteria [2, 3]. Before ablation, operators opened the PANO View to expose the endocardial surface of the heart chambers, for distinguishing the special anatomic landmarks. PANO View is an unfolded projection of the chamber for the operators to visualize the anatomy of intracardiac structures. During ablation, operators could monitor real-time TP and achieve a TP between 3 and 30 g to get an effective lesion and avoid complications.

#### RFCA procedure of AT/AFL

Activation mapping of the AT were created using a non-irrigated ablation catheter (Triguy; APT Medical, CHN). Focal mechanism was confirmed if activation mapping showing centrifugal activation from a central source. RFCA was performed at the site of earliest activation. For patients suspected AFL, high-resolution mapping was performed using a multielectrode mapping catheter (Triguy; APT Medical, CHN). The diagnosis of macro-reentrant AT was based on the continuous activation mapping pattern with the “early meets late” pattern and the whole activation time of the target atrium exceeds 90% of the tachycardia cycle length (TCL) [4]. Additionally, entrainment mapping was also used to confirm the reentrant circuit only for patients with stable TCL. Sites were confirmed inside the circuit if the difference between the postpacing interval and TCL was < 30 ms. After confirming the reentrant circuit, PANO View was opened to distinguish the pouch, the cavo-tricuspid isthmus, and other anatomic landmarks to design the ablation strategies (Fig. 3). An irrigated ablation catheter (Triguy; APT Medical, CHN) was used to perform ablation of the critical isthmus.

#### RFCA procedure of AVNRT

For patients with AVNRT, the stepwise ablation process was as follows. First, the ablation catheter was advanced into the heart, and an instant image of the right atrium was roughly created. Second, a higher resolution image was created as the ablation catheter was navigated through and contacted the main structures, including the CS and the tricuspid annulus. Then, the operator would use the right anterior oblique (RAO) projection of the 3D reconstruction model to open the PANO View. Careful adjustment of the cutting plane ascertained a clear view of the Koch’s triangle and the slow-pathway (SP) region. Finally, RF was delivered at the SP region identified by the PANO View.

#### RFCA procedure of AVRT

For patients with AVRT, a non-irrigated ablation catheter (Triguy; APT Medical, CHN) was used to set up the activation mapping during tachycardia or ventricular pacing and to reproduce the annuli under point-by-point module. RF energy was delivered at the sites with one of following features: (1) earliest retrograde A-wave during orthodromic AVRT or ventricular pacing; (2) earliest local V-wave proceeding the delta wave during sinus rhythm.

### RFCA procedure of AF

Oral anticoagulation was continued for at least four weeks before and three months after ablation. RFCA mainly consisted of antral PV isolation (PVI) and was performed under sedation using midazolam and fentanyl. After transseptal puncture, 3D reconstruction of the left atrium (LA) was generated using the KODEX-EPD system with a steerable PV mapping catheter (Triguy; APT Medical, CHN). Ablation was performed with an irrigated ablation catheter (Triguy; APT Medical, CHN) through the right femoral vein to obtain a contiguous lesion set for ipsilateral circumferential PVI. The radiofrequency power ranged from 35–40 W. The ablation target time were 30 s anterior and 20 s for posterior. Verification of entrance block was performed for all PVs with the steerable PV mapping catheter after a twenty-minute waiting time. Additionally, for patients diagnosed with paroxysmal AF, 40 mg adenosine triphosphate (ATP) and isoproterenol were given to verify the block of PVs and induce AF.

### Cryoablation procedure of AF

In patients who underwent with cryoablation for AF, a single transeptal puncture was performed and an 8.5 F sheath (St. Jude Medical, Inc, St Paul, MN, USA) was introduced into the LA. Selective PV-targeted angiography was perform to identify the PV ostia individually. The 8.5F sheath was changed to a 15F steerable sheath (Flexcath Advance, Medtronic, Inc.). Then, a 28 mm cryoballoon (CB) catheter (Arctic Front Advance, Medtronic, Inc.) and a 20 mm circular mapping catheter (Achieve; Medtronic, Inc.) were introduced into LA. The 3D reconstruction of LA and PVs was performed by the KODEX-EPD system. After positioning the mapping catheter into the PV ostium to record PV potentials, the CB catheter was inflated. At the same time, the KODEX occlusion tool software compared the baseline impedance recorded by a circular catheter inside the PV ostium with the impedances recorded when the balloon was inflated. The comparison was performed for each of the eight electrodes of the circular catheter, and the results were used to evaluate the occlusion degree: the green electrode indicates good occlusion, while the red electrode indicates a gap in the occlusion. In this way, in the case of partial occlusion shown by more than one red electrode, operators could adjust the balloon to obtain complete occlusion without dye injections and angiography. After achieving complete occlusion, as shown by all green electrodes, contrast medium injections through the inflated CB were used to verify complete occlusion. Cryoablation was started with the isolation of the left superior PV (LSPV), followed by the left inferior PV (LIPV), right superior PV (RSPV), and right inferior PV (RIPV). To avoid phrenic nerve injury, real-time monitoring of phrenic nerve function was performed by continuous phrenic nerve pacing when cryoablation was performed on right PVs. The target cryoablation time was 90–180 s.

### Follow-up

Patients who underwent ablation were followed up by outpatient visits or telephone calls. For patients with AF, the outpatient clinic follow-up was regularly at 3, 6, and 12 months and then every 6 months thereafter or whenever symptoms occurred. All patients underwent 12-lead ECG during each clinic visit, and 24 h Holter was recorded at 3 and 6 months and every 6 months. All arrhythmia-related symptoms, including palpitations, chest tightness, chest pain, dyspnea, and syncope were recorded. A 12-lead ECG or 24 h Holter was assessed when patients complained of any of the symptoms mentioned. The final censor date for this study was April 20th, 2022.

### Data analysis

Statistical analyses were performed using SPSS IBM 22 (IBM Co., Armonk, New York, USA). GraphPad Prism 8.0 (GraphPad Software Inc., La Jolla, CA) was used for graphing. Continuous data are presented as means ± standard deviations (SDs) or medians and interquartile ranges (25^th^-75^th^ percentiles), depending on the normality of the distribution. Categorical variables were described as frequency counts and percentages. For continuous data, either Student’s two-tailed *t test* or the Mann-Whiney *U test* was performed for statistical comparisons. For comparisons of categorical data, the chi-square test was performed. Kaplan–Meier curves were generated, and log rank *p value* was calculated in the survival analysis. A probability value of *p* < 0.05 indicated statistical significance.

## Results

### Baseline characteristics

**Fig. 4:**
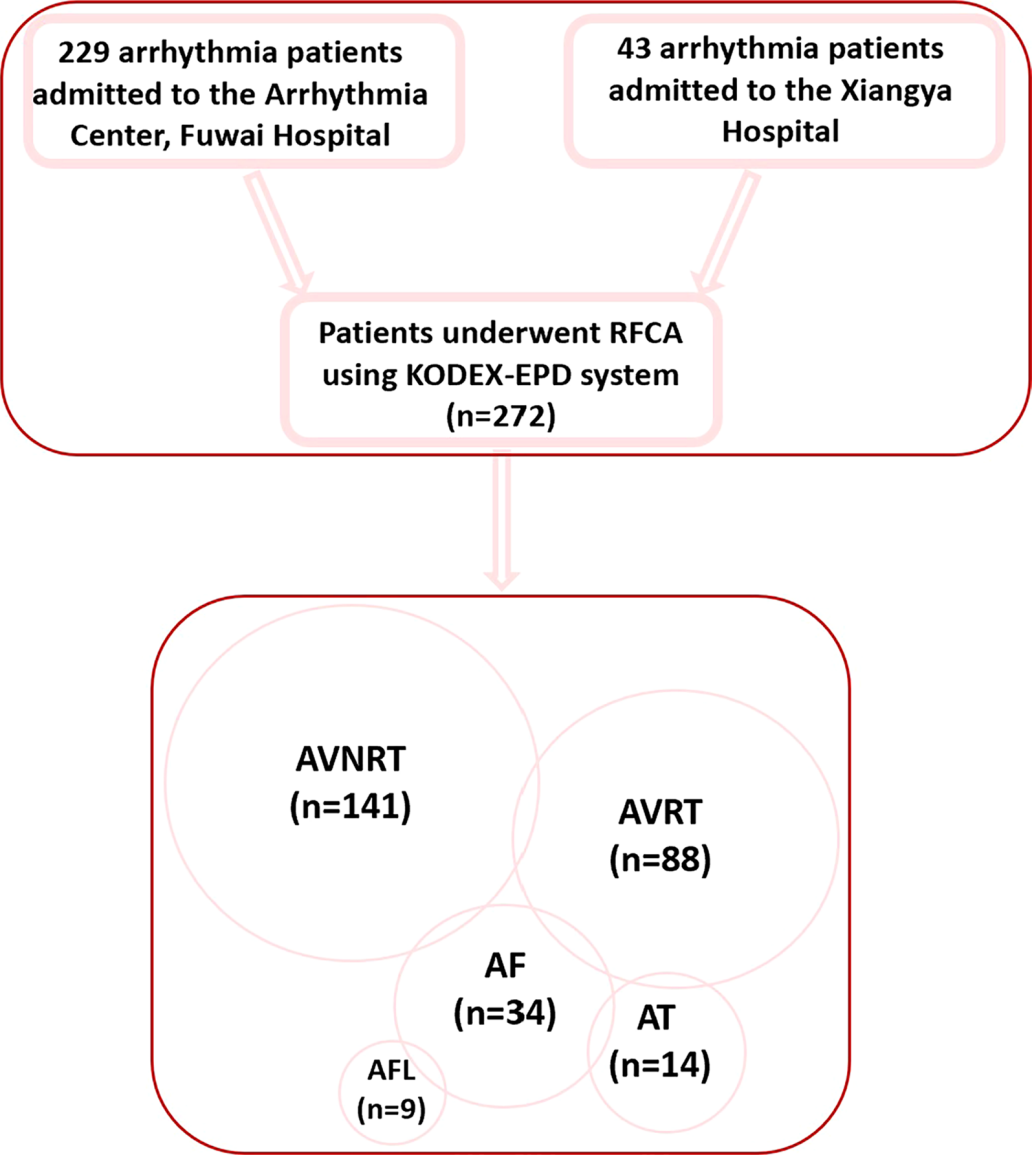
Flow chart of all patients who underwent RFCA using the KODEX-EPD system. AF, atrial fibrillation; AFL, atrial flutter; AT, atrial tachycardia; AVRT, atrioventricular reentrant tachycardia; AVNRT, atrioventricular nodal reentrant tachycardia; PVC, premature ventricular contraction; RFCA, radiofrequency catheter ablation

A total of 272 subjects were enrolled from October 2020 to July 2021. Of them, 229 patients were enrolled in Fuwai Hospital, and 43 patients were enrolled in Xiangya Hospital (Fig. 4). Figure 4 also shows the distribution of all arrhythmias. Of the 272 enrolled patients, 262 patients had only one kind of arrhythmia, and 10 patients were diagnosed with more than one kind of arrhythmias. Specifically, in all populations, 15 (5.5%) patients had AT, 88 (32.4%) had AVRT, 141 (51.8%) had AVNRT, 34 (12.5%) had AF, and 9 (3.3%) had AFL. All AF patients included were first-do-PVI. Of all the AF patients, there were 26 paroxysmal AF and 8 persistent AF. No comparisons were performed because of the relatively small size in each subgroup. Table 1 summarizes the subject demographics. Of 272 patients, there was no sex preponderance in all subgroups, except for the AT and AF groups. The overall median age was 47.2 ± 18.1 years. Patients with AF or AFL were older than other subgroups. Ten (3.6%) patients experienced previous ablation and mainly gathered in AVRT and AVNRT patients. A total of 3 (3.4%) patients with AVRT, 6 (4.3%) with AVNRT, and 1 (2.9%) with AF had an ablation history before this admission. The AF patient with an ablation history was ablated for AVNRT in the past.

**Table 1:** Demographic and clinical characteristics

Parameters	All cases (n, 272)	AT (n, 15)	AVRT (n = 88)	AVNRT (n, 141)	AFL (n, 9)	AF (n, 34)
Male gender, n (%)	133 (48.9)	9 (60.0)	42 (47.7)	63 (44.7)	4 (44.4)	24 (70.6)
Age, year	47.2 ± 18.1	50.4 ± 16.7	44.6 ± 17.6	45.5 ± 19.0	62.1 ± 9.2	56.8 ± 11.4
Weight, kg	68.0 ± 13.8	77.3 ± 18.0	64.3 ± 12.4	67.5 ± 13.5	70.8 ± 8.8	76.4 ± 12.4
Height, cm	166.9 ± 10.2	171.5 ± 9.9	166.9 ± 12.4	165.9 ± 11.6	167.6 ± 9.2	169.3 ± 7.8
BSA, m^2^	1.9 ± 0.2	2.0 ± 0.3	1.8 ± 0.2	1.8 ± 0.2	1.9 ± 0.2	2.0 ± 0.2
BMI, kg/m^2^	24.4 ± 4.9	26.2 ± 4.4	23.0 ± 3.3	24.7 ± 5.9	25.2 ± 1.7	26.5 ± 2.9
Duration from symptoms onset to first EPS (months)	78.9 ± 108.1	44.1 ± 63.0	90.4 ± 125.1	80.3 ± 105.5	36.4 ± 76.9	50.6 ± 67.7
Previous ablation (n, %)	10 (3.7)	0 (0)	3 (3.4)	6 (4.3)	0(0)	1 (2.9)
Comorbidities, n (%)						
HTN, n (%)	47 (17.3)	1 (6.7)	13 (14.8)	19 (13.5)	4 (44.4)	11 (32.4)
CAD, n (%)	28 (10.3)	2 (13.3)	6 (6.8)	17 (12.1)	1 (11.1)	4 (11.8)
DM, n (%)	23 (8.5)	4 (26.7)	5 (5.7)	9 (6.4)	3 (33.3)	6 (17.6)
CHD, n (%)	7 (2.6)	0 (0)	0 (0)	4 (2.8)	2 (22.2)	1 (2.9)
VHD, n (%)	8 (2.9)	0 (0)	0 (0)	4 (2.8)	3 (33.3)	2 (5.9)
PH, n (%)	3 (1.1)	1 (6.7)	0 (0)	2 (1.4)	1 (11.1)	0 (0)
NYHA-FC, n (%)						
I/II	267 (98.2)	14 (93.3)	87 (98.9)	137 (97.2)	8 (88.9)	34 (100)
III/IV	5 (1.8)	1 (6.7)	1 (1.1)	4 (2.8)	1 (11.1)	0 (0)

AF, atrial fibrillation; AFL, atrial flutter; AT, atrial tachycardia; AVRT, atrioventricular reentrant tachycardia; AVNRT, atrioventricular nodal reentrant tachycardia; BSA, body surface area; BMI, body mass index; CAD, coronary artery disease; CHD, congenital heart disease; DM, diabetes mellitus; EPS, electrophysiology study; HTN, hypertension; PH, pulmonary hypertension; PVC, premature ventricular contraction; VHD, valvular heart disease; NYHA-FC, New York Heart Association functional class

The most common comorbid condition was hypertension (17.3%), followed by coronary heart disease (CAD) (10.3%), and diabetes mellitus (DM) (8.5%). Three patients (1.1%) had a history of pulmonary hypertension and were diagnosed with right heart catheterization. Of them, two patients were diagnosed with AVNRT. Over ninety-five percent (98.2%) of patients were in New York Heart Association functional class I/II, and only 5 (1.8%) were in class II/IV.

Table 2 shows the echocardiography parameters. All patients had normal ejection fractions. Medical therapy in the AF group is summarized in Additional file 1: Table 1. Beta-blocker (16/47.0%) was the most common antiarrhythmic medicine used in the AF subgroup to control heart rate and rhythm. Nine patients (26.5%) and seven patients (20.6%) were taking amiodarone and dronedarone after AF ablation, respectively. Nearly 95% percent of AF patients had CHA_2_DS_2_-VASc scores of more than two points, and 29 patients were treated with Rivaroxaban.

**Table 2:** Echocardiography and procedure parameters

Parameters	All cases (n, 272)	AT (n, 15)	AVRT (n = 88)	AVNRT (n, 141)	AFL (n, 9)	AF (n, 34)
LA dimension (AP), mm	33.6 ± 5.6	35.3 ± 5.9	32.1 ± 4.5	32.7 ± 4.9	41.3 ± 7.6	40.1 ± 4.5
LVEDD, mm	45.8 ± 4.6	46.6 ± 3.5	45.1 ± 4.0	45.2 ± 4.4	49.8 ± 7.9	49.1 ± 5.0
Ejection fraction, %	64.2 ± 6.2	66.8 ± 4.9	64.0 ± 7.4	64.7 ± 5.6	60.3 ± 10.6	61.9 ± 7.1
RV dimension (AP), mm	23.9 ± 5.3	28.1 ± 4.7	23.5 ± 5.2	23.3 ± 5.1	26.6 ± 2.6	26.0 ± 6.0
Mapping time, minutes	8.5 ± 4.5	15.3 ± 8.7	9.3 ± 5.1	5.9 ± 5.8	15.8 ± 4.5	10.5 ± 3.1
Procedure time, minutes	81.9 ± 31.0	72.5 ± 16.7	69.2 ± 9.9	63.8 ± 9.1	76.5 ± 9.3	132.7 ± 19.6
Fluoroscopy time, seconds	273.8 ± 327.4	100.3 ± 85.3	93.4 ± 78.3	85.2 ± 65.4	246.5 ± 96.4	480.3 ± 122.3
Ablation time, seconds	522.5 ± 585.4	295.0 ± 126.7	295.9 ± 128.2	296.1 ± 167.6	428.9 ± 61.3	1830.2 ± 484.6
RFCA applications	23.2 ± 76.9	6.7 ± 4.6	6.4 ± 3.4	5.8 ± 3.1	14.1 ± 2.0	53.5 ± 33.6
Immediate success, n (%)	272 (100.0)	15 (100.0)	88 (100.0)	141 (100.0)	9 (100.0)	34 (100)
Complications, n (%)	1 (0.4)	0 (0)	0 (0)	1 (0.7)	0 (0)	0 (0)
Death, n (%)	0 (0)	0 (0)	0 (0)	0 (0)	0 (0)	0(0)
Pericardial effusion or tamponade, n (%)	0 (0)	0 (0)	0 (0)	0 (0)	0 (0)	0(0)
Myocardial infarction, n (%)	0 (0)	0 (0)	0 (0)	0 (0)	0 (0)	0(0)
Stroke or TIA, n (%)	0 (0)	0 (0)	0 (0)	0 (0)	0 (0)	0(0)
Vascular complications, n (%)	0 (0)	0 (0)	0 (0)	0 (0)	0 (0)	0(0)
II-III degree AVB, n (%)	0 (0)	0 (0)	0 (0)	0 (0)	0 (0)	0 (0)
PE, n (%)	1 (0.4)	0 (0)	0 (0)	1 (0.7)	0 (0)	0 (0)
Phrenic nerve palsy, n (%)	0 (0)	0 (0)	0 (0)	0 (0)	0 (0)	0(0)
Atrioesophageal fistula, n (%)	0 (0)	0 (0)	0 (0)	0 (0)	0 (0)	0(0)
PV stenosis, n (%)	0 (0)	0 (0)	0 (0)	0 (0)	0 (0)	0(0)
Recurrence, n (%)	10 (3.7)	0 (0)	1 (1.1)	2 (1.4)	1 (11.1)	6 (27.6)

AF, atrial fibrillation; AFL, atrial flutter; AT, atrial tachycardia; AVB, atrioventricular block; AVRT, atrioventricular reentrant tachycardia; AVNRT, atrioventricular nodal reentrant tachycardia; LA, left atrial; AP, anteroposterior; LVEDD, left ventricular end-diastolic dimension; PE, pulmonary embolism; PV, pulmonary vein; RV, right ventricle; TIA, transient ischemic attack

### Procedure data

Procedure data are shown in Table 2. All ATs were confirmed as focal AT and had only one origin. Eight of them originated from the coronary sinus ostium, two from the right atrial appendage and five from the crista terminalis. Besides that, eight of AFL patients were diagnosed as antidromic cavo-tricuspid isthmus dependent AFL and one was diagnosed as orthodromic cavo-tricuspid isthmus dependent AFL. The mapping time in AT, AVRT, AVNRT, AFL, and AF were 15.3 ± 8.7 min, 9.3 ± 5.1 min, 5.9 ± 5.8 min, 15.8 ± 4.5 min, and 10.5 ± 3.1 min, respectively (Table 2). The AVRT group (69.2 ± 9.9 min) and AVNRT group (63.8 ± 9.1 min) had the shortest procedure time. The AF group (132.7 ± 19.6 min) had the longest procedure time, and the AT and AFL groups had the longest mapping times (15.3 ± 8.7 min and 15.8 ± 4.5 min, respectively). For the ablation time and RFCA applications, the AT, AVNRT, and AVRT subgroups were less than the other subgroups (295.0 ± 126.7 s and 6.7 ± 4.6; 295.9 ± 128.2 s and 6.4 ± 3.4; 296.1 ± 167.6 s and 5.8 ± 3.1, respectively).

Among all AF patients, seven (20.6%) underwent PVI using a 28 mm cryoballoon. The mean numbers of cryoablation applications per PV, except bonus freeze, were as follows: LSPV, 2.1 ± 0.4; LIPV, 2.3 ± 0.5; RSPV, 2.4 ± 0.8; and RIPV, 1.6 ± 0.9. The mean temperature reached during the procedure per PV was as follows: -49.0 ± 5.4 ℃ for LSPV, -40.0 ± 4.3 ℃ for LIPV, -48.0 ± 5.9 ℃ for RSPV, and -39.2 ± 7.9 ℃ for RIPV. Other details of cryoablation ablation data are shown in Additional file 1: Table 2. Table 3 shows comparisons between RFCA and cryoablation of AF. In both groups, most patients had paroxysmal AF (20/26 vs. 6/7). Patients who underwent cryoablation had shorter procedure times, ablation times, and ablation applications than the RFCA group (117.2 ± 16.8 min vs. 137.5 ± 18.2 min; 1537.7 ± 537.9 s vs. 1933.0 ± 397.5 s; and 8.9 ± 2.9 vs. 72.6 ± 15.1, respectively).

**Table 3:** Procedure details of AF

	All (n-34)	RFCA (n = 27)	Cryoablation (n = 7)
AF type, n (%)			
Paroxysmal AF, n (%)	26 (76.5)	20 (74.1)	6 (85.7)
Persistent AF, n (%)	8 (23.5)	7 (25.9)	1 (14.3)
Ablation catheter	–	Irrigated ablation catheter (Triguy, APT Medical, Shenzhen, CHN)	Cryoballon catheter (Arctic Front Advance, Medtronic, Inc.)
Mapping catheter	–	Steerable PV mapping catheter (Triguy, APT Medical, Shenzhen, CHN)	Inner lumen circular mapping catheter (Archieve; Medtronic, Inc.)
Procedure time, minutes	132.7 ± 19.6	137.5 ± 18.2	117.2 ± 16.8
Ablation time, seconds	1830.2 ± 484.6	1933.0 ± 397.5	1537.7 ± 537.9
Ablation applications	53.5 ± 33.6	72.6 ± 15.1	8.9 ± 2.9
Recurrence, n (%)	6 (17.6)	5 (18.5)	1 (14.3)

AF, atrial fibrillation; PV, pulmonary vein; RFCA, radiofrequency catheter ablation

### Outcomes and survival analysis

**Fig. 5:**
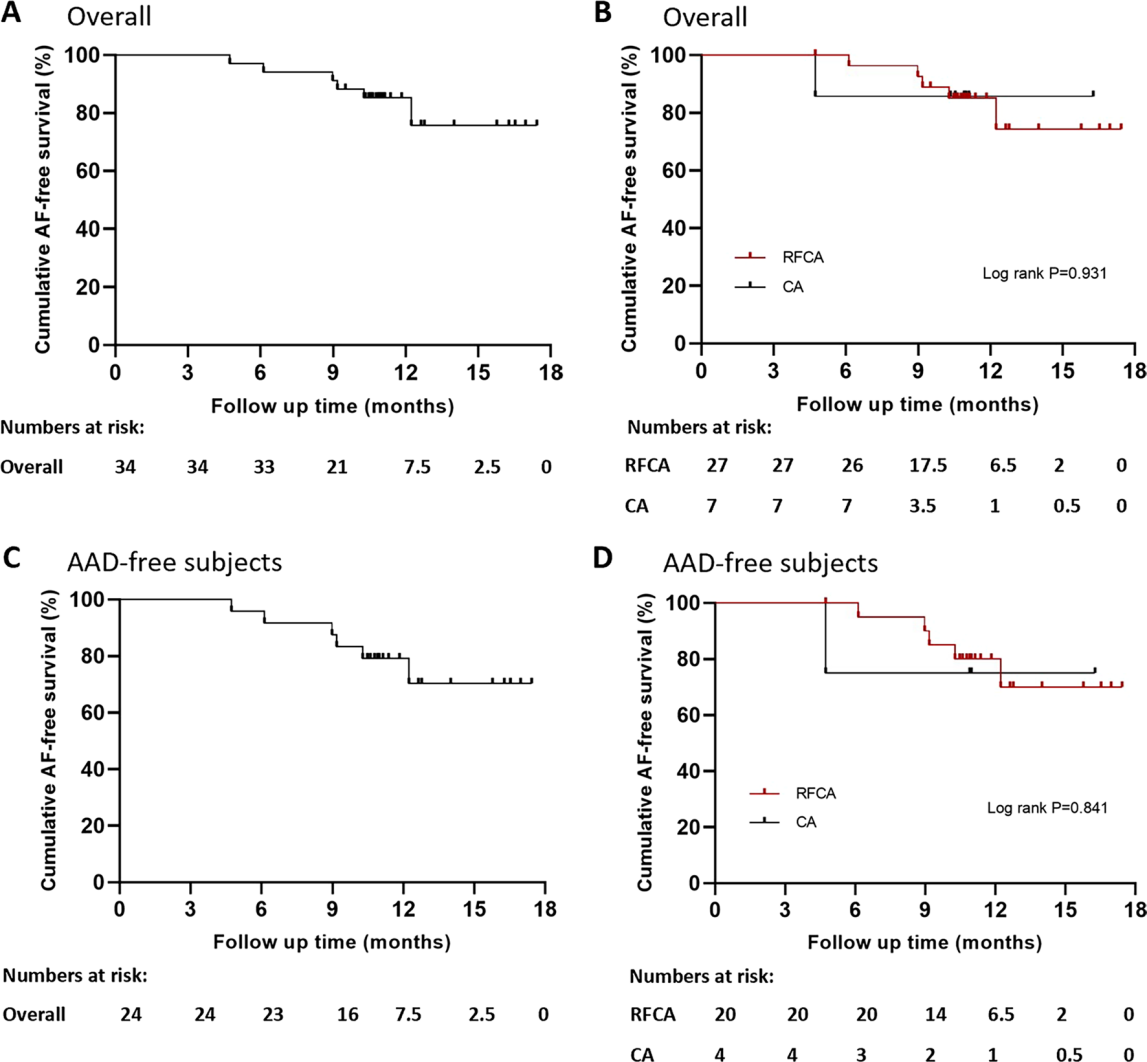
Freedom from recurrent AF of **A** all AF patients who underwent ablation; **B** patients with AF who underwent ablation divided by RFCA or CA with AADs. Freedom from recurrent AF of **C** AF patients who underwent ablation and **D** patients with AF who underwent ablation divided by RFCA or CA without AADs. AADs, antiarrhythmic drugs; AF, atrial fibrillation; CA, cryoablation; RFCA, radiofrequency catheter ablation

All patients achieved immediate ablation success. The mean follow-up duration of our population was 11.8 ± 2.4 months. There was no recurrence in the AT subgroup. One patient (1.1%) in the AVRT subgroup and two patients (1.4%) in the AVNRT subgroup experienced recurrence. Among AF patients, three patients had early AF recurrence within the first three months of follow-up after ablation. When considering a three-month blanking time, the estimated freedom of AF at one-year after ablation with and without AADs was 75.7% and 70.4%, respectively (six patients experienced recurrence after three-months of blanking; Fig. 5A, C). The Kaplan − Meier analysis showed no significant difference in the overall AF recurrence (log-rank; P, 0.931) or AAD-free AF recurrence (log-rank; P, 0.841) between RFCA and cryoablation (Fig. 5B, D).

Regarding complications, only one patient with AVNRT (0.4%) had mild pulmonary embolism the day after the ablation procedure, which resolved after three months of anticoagulation therapy. None of the patients died or had a cerebrovascular event in the periprocedural period.

## Discussion

The present study illustrated the safety and effectiveness of ablation using the novel wide-band dielectric mapping system in two Chinese centers. The major finding is as follows: RFCA and cryoablation using the novel wide-band dielectric mapping system are safe, feasible, and effective in common tachyarrhythmias.

The novel 3D-mapping system, the KODEX-EPD system, has come onto the market in recent years [5]. In this study, we evaluated the utility of this novel mapping technology, which offers several potential advantages for mapping and ablating tachyarrhythmias. These include: (1) a novel algorithm to create high-resolution images of cardiac anatomy by distinct dielectric properties of different tissues; (2) the PANO View, offering a depiction of the endocardial surface within a single image to help differentiate the anatomic landmarks and design ablation lines; and (3) the occlusion tool software, which is equipped into the mapping system, could assess PV occlusion by the cryoballoon catheter.

In our population, AVNRT, AVRT, and AF were the most common tachyarrhythmias. The immediate success rate of RFCA for AVNRT and AVRT was 100%, and the long-term success rates were 98.9% and 98.6%, respectively. Meanwhile, only one mild pulmonary embolism occurred, and no major complications were observed. These results were comparable with other previous studies [6, 7]. Chrispin et al. [6] included 743 patients with AVNRT who underwent RFCA using the CARTO system (Biosense Webster, Diamond Bar, California, USA) or Ensite system (St. Jude Medical, St Paul, Minnesota, USA). They reported that acute and long-term success were 98.5% and 95.5%, respectively. They also found that major complications occurred in nine patients (1.2%), including pericardial effusion, vascular complications, and a high degree atrioventricular block. According to our experience, RFCA of SVT using KODEX-EPD have serval advantages over the other mapping systems. First, the PANO View of KODEX-EPD system helps to reduce the exposure of X-ray. Second, the PANO View helps the operator directly distinguish the Koch’s triangle, the slow-pathway region, the atrioventricular annuli, and the cavo-tricuspid isthmus to get a more efficient ablation of the target region. Third, the KODEX-EPD system could monitor real-time CF using non-CF catheters without increases expenses.

Several prior studies have reported the long-term AF-free success of RFCA. The TOCCASTAR trial [8] studied symptomatic, paroxysmal AF using the TactiCath CF catheter and the EnSite NavX electroanatomic mapping system (St. Jude Medical); they found that the long-term success rate of freedom from AF, AT, or AFL at 12 months was 67.8%. The next generation of TactiCath CF catheters achieved 82.2% AF success at one year [9]. In the SMART AF trial [10], the drug-free 1-year success rate reported was 65.8%. RADAR is another novel 3D system, and Choudry et al. [11] included four centers. Sixty-four patients with AF underwent RFCA using RADAR. After 12.6 ± 0.8 months of follow-up, 68.0% remained AF-free off all antiarrhythmics. The estimated AF-free success (75.7%) at one-year reported in our study compares favorably with the reported trials. However, it is difficult to directly compare our results with other studies owing to significant differences in ablation targets and protocol design. Regarding safety, the TOCCASTAR study [8] resulted in a cardiac tamponade rate of 0.66% and the SMART SF trial [10] reported a primary adverse event rate of 2.5% (including cardiac tamponade, thromboembolism and transient ischemic attack). No deaths, stroke, cardiac tamponade, or other complications were reported in our AF population. This finding could have occurred due to the relatively small sample size and the use of the PANO View, which helps to design the ablation lines in the endocardial surface (Fig. 2). Further randomized studies on AF ablation using the KODEX-EPD system are needed.

KODEX-EPD is a novel wide-band dielectric imaging system that creates high-resolution images of cardiac anatomy based on distinct dielectric tissue properties, which is different from other mapping systems. PANO View provides a clear visualization of the endocardial surface, helps design the ablation lines, and advances the orientation for educational purposes. Many researchers have already used the PANO View to determine the anatomic landmarks of the LA [5, 12]; thus, this approach will be used for guidance of transseptal puncture, catheter advancement, and ablation of other complex arrhythmias in the future. Meanwhile, TP technology has already integrated within the KODEX-EPD system, which can achieve real-time monitoring of CF with currently available non-CF catheters. Soon, the KODEX-EPD system will continue to include wall thickness and ablation response assessment functionalities. With the integration of precise high-resolution imaging, compatible real-time CF monitoring and wall thickness as well as tissue response diagnostics, the KODEX-EPD system has the potential to multidimensionally guide mapping and ablation of more complex arrhythmias.

## Limitations

There are several limitations to this study. First, it is a heterogeneous population composed of different supraventricular tachycardia patients and a relatively small number of AF patients. Second, this study was a retrospective study without any control group, and a multicenter, prospective study with a larger AF population compared with a control group should be performed to illustrate the safety and effectiveness of the KODEX-EPD system. Despite these limitations, the initial outcomes are favorable in catheter ablation of tachyarrhythmias using the KODEX-EPD system.

## Conclusions

This retrospective, two-center study demonstrated that catheter ablation of supraventricular arrhythmias using the KODEX-EPD system is feasible, safe, and effective.

## Supplementary Information

**Additional file 1. Supplementary Table 1**. Medical therapy data in AF group.** Supplementary Table 2**. Cryoballoon ablation data.

## Data Availability

The datasets presented in this article are not readily available because research data is confidential. Data sharing requests are required to meet the policies of the hospital and the funder. Requests to access the datasets should be directed to doctortangmin@yeah.net.
