# Supplementary material for: Safety and effectiveness of a novel dielectric mapping system: one-year, two chinese centers experiences
Source: BMC Cardiovasc Disord. 2022 Aug 3;22:352. doi: 10.1186/s12872-022-02790-8 (PMC9351078; doi:10.1186/s12872-022-02790-8)
Supplement: Supplementary file 1 — Additional file 1. Supplementary Table 1. Medical therapy data in AF group. Supplementary Table 2. Cryoballoon ablation data. [file 12872_2022_2790_MOESM1_ESM.docx]

**Supplementary Table 1 Medical therapy data in AF group**

| **Variables** | **AF group (n=34)** |
| --- | --- |
| Antiarrhythmic treatment after the procedure until third month |  |
| Beta-blocker | 16 (47.0) |
| Dronedarone | 7 (20.6) |
| Amiodarone | 9 (26.5) |
| Propafenone | 2 (5.9) |
| CHA2DS2-VASc score ≥2, n (%) | 32 (94.1) |
| Anticoagulation therapy |  |
| Warfarin | 1 (2.9) |
| Rivaroxaban | 29 (85.3) |
| Dabigatran | 3 (8.9) |
| Edoxaban | 1 (2.9) |

**Supplementary Table 2 Cryoballoon ablation data**

| **Variables** | **AF patients (n=7)** |
| --- | --- |
| LSPV |  |
| Minimum temperature, ℃ | -49.0 ± 5.4 |
| Ablation time, seconds | 377.1 ± 75.2 |
| Mean number of applications | 2.1 ± 0.4 |
| LIPV |  |
| Minimum temperature, ℃ | -40.0 ± 4.3 |
| Ablation time, seconds | 402.9 ± 96.2 |
| Mean number of applications | 2.3 ± 0.5 |
| RSPV |  |
| Minimum temperature, ℃ | -48.0 ± 5.9 |
| Ablation time, seconds | 420.0 ± 158.7 |
| Mean number of applications | 2.4 ± 0.8 |
| RIPV |  |
| Minimum temperature, ℃ | -39.2 ± 7.9 |
| Ablation time, seconds | 325.0 ± 134.7 |
| Mean number of applications | 1.6 ± 0.9 |
